# Improving variant calling by incorporating known genetic variants into read alignment

**DOI:** 10.1186/1471-2105-16-S15-P18

**Published:** 2015-10-23

**Authors:** Nam S Vo, Vinhthuy Phan

**Affiliations:** 1 Department of Computer Science, University of Memphis, Memphis, TN 38152, USA

## Background

The identification of genetic variants has great significance in genetic research. To call variants using next-generation sequencing data, current methods rely primarily on mapped reads produced by a separate read aligner without taking into account existing genetic variants[1]. Thus, these methods usually require a large number of reads (high coverage) to be able to detect variants accurately[2]. Moreover, the separation of read alignment and variant calling results in a workflow is complex and involves many separate steps and different tools[3].

## Materials and methods

We introduce a novel method that leverages existing information about genetic variants to improve performance of variant calling. Incorporating known variants allows reads to be aligned more accurately and variants to be detected accurately with low coverage. This method further integrates two separate processes of read alignment and variant calling into one unified workflow, which results in a much more automated, simplified and faster process. A Bayesian method is used to calculate quality of variant calls.

## Results

We showed that this method significantly improved the accuracy of variants on simulated data on human chromosomes, especially with low-coverage data, compared to popular methods such as GATK. At low coverage (<= 5x), this method achieved recall rates that were 2-19% higher while maintaining competitive precision compared to GATK. In particular, the method showed a significant improvement on identifying INDELs with recall rates 33-42% higher than GATK, and precision rates 9-34% higher than GATK. Our method also simplifies the workflow greatly, requiring 2 steps to call variants while GATK requires 6-8 steps and 2 external tools including Picard and SAMtools to preprocess the data.

## Conclusions

As genetic variants are being collected for more and more people, the integration of existing information into the calling of variants is realistic. We demonstrated that by incorporating existing variant information, accurate detection of variants could be achieved even with low coverage. Thus, the method is promising in helping to reduce experimental cost.

## Availability

Source code and testing data are available at https://github.com/namsyvo/IVC.
